# Ultra high pressure homogenization (UHPH) inactivation of *Bacillus amyloliquefaciens* spores in phosphate buffered saline (PBS) and milk

**DOI:** 10.3389/fmicb.2015.00712

**Published:** 2015-07-14

**Authors:** Peng Dong, Erika S. Georget, Kemal Aganovic, Volker Heinz, Alexander Mathys

**Affiliations:** ^1^College of Food Science and Nutritional Engineering, China Agricultural UniversityBeijing, China; ^2^National Engineering Research Center for Fruits and Vegetables ProcessingBeijing, China; ^3^Key Laboratory of Fruits and Vegetables Processing, Ministry of AgricultureBeijing, China; ^4^German Institute of Food TechnologiesQuakenbrück, Germany; ^5^Institute of Food Chemistry, Leibniz Universität HannoverHannover, Germany

**Keywords:** ultra high pressure homogenization, bacterial spore, *Bacillus amyloliquefaciens*, inactivation, milk, fat content

## Abstract

Ultra high pressure homogenization (UHPH) opens up new areas for dynamic high pressure assisted thermal sterilization of liquids. *Bacillus amyloliquefaciens* spores are resistant to high isostatic pressure and temperature and were suggested as potential surrogate for high pressure thermal sterilization validation. *B. amyloliquefaciens* spores suspended in PBS buffer (0.01 M, pH 7.0), low fat milk (1.5%, pH 6.7), and whole milk (3.5%, pH 6.7) at initial concentration of ~10^6^ CFU/mL were subjected to UHPH treatments at 200, 300, and 350 MPa with an inlet temperature at ~80°C. Thermal inactivation kinetics of *B. amyloliquefaciens* spores in PBS and milk were assessed with thin wall glass capillaries and modeled using first-order and Weibull models. The residence time during UHPH treatments was estimated to determine the contribution of temperature to spore inactivation by UHPH. No sublethal injury was detected after UHPH treatments using sodium chloride as selective component in the nutrient agar medium. The inactivation profiles of spores in PBS buffer and milk were compared and fat provided no clear protective effect for spores against treatments. Treatment at 200 MPa with valve temperatures lower than 125°C caused no reduction of spores. A reduction of 3.5 log_10_CFU/mL of *B. amyloliquefaciens* spores was achieved by treatment at 350 MPa with a valve temperature higher than 150°C. The modeled thermal inactivation and observed inactivation during UHPH treatments suggest that temperature could be the main lethal effect driving inactivation.

## Introduction

Bacterial spores pose a major hazard in food safety because of their high resistance to most hurdles. Thermal sterilization is an effective method to inactivate bacterial spores, however, it has negative effects on sensorial and nutritional qualities of foods (Reineke et al., 2013; Georget et al., 2014b). Hence, alternative preservation technologies have been investigated to inactivate bacterial spores while retaining sensorial and nutritional properties, such as high isostatic pressure (Mathys et al., 2007a; Reineke et al., 2011a, 2013; Georget et al., 2015), pulsed electric fields (Siemer et al., 2014a,b; Toepfl et al., 2014), ultra-violet light (Baysal et al., 2013; Gayán et al., 2013), and ultra-high pressure homogenization (UHPH) (Georget et al., 2014a,b). Conventional homogenizers with pressures up to 50 MPa have been used in beverages, pharmaceutical and cosmetic industries to reduce particle size and produce stable emulsions but are not sufficient to induce bacterial spore inactivation. It is suspected that this is due to the low homogenization pressure and the resulting low valve temperatures. The recent enhancement of homogenization pressure up to 400 MPa, within which pressure range >200 MPa can be regarded as UHPH, has opened up new areas for dynamic pressure assisted thermal sterilization of liquids (Diels and Michiels, 2006; Georget et al., 2014b). UHPH is a continuous process that has potential application in the processing of liquid foods including juices, vegetable milk, and dairy products. The inactivation of microorganisms is achieved by UHPH and thus the shelf-life can be extended. The products are of improved sensorial and nutritional characteristics, among others, because of the increased emulsion stability and very short residence time at high pressure and/or high temperature (Zamora and Guamis, 2015).

Inactivation of different bacterial spores in model and food systems by UHPH has been reviewed and the overview highlights a varying success (Georget et al., 2014b). The naturally present spores in milk with 3.5% fat decreased from 1.7 to 0.6 log_10_CFU/mL after treatment at 300 MPa with valve temperature at 103°C (Pereda et al., 2007). For *Geobacillus stearothermophilus* ATCC7953 and *Clostridium sporogenes* PA3679 spores in skim milk, a UHPH treatment with 16 passes at 300 MPa with an inlet temperature at 45°C caused a reduction of only 0.67 log_10_CFU/mL (Pinho et al., 2011). However, several successful investigations were also reported. It was found that up to 5 log_10_CFU/mL *Bacillus subtilis* PS832 and 2 log_10_CFU/mL *G. stearothermophilus* ATCC7953 spores suspended in phosphate buffered saline (PBS buffer) could be inactivated by single pass UHPH treatments applying pressures higher than 300 MPa and valve temperatures higher than 145°C with estimated holding times below 0.5 s. It was concluded that the very high valve temperatures could be a dominant parameter leading to bacterial spores' inactivation (Georget et al., 2014a). In another investigation, commercial whole milk with 3.6% fat was inoculated with *Bacillus cereus, Bacillus licheniformis, Bacillus sporothermodurans, Bacillus coagulans, G. stearothermophilus*, and *B. subtilis* spores. UHPH treatments at 300 MPa with inlet temperature at 75 and 85°C were capable of a reduction of 5 log_10_CFU/mL (Amador Espejo et al., 2014). These recent investigations suggest a high potential of UHPH for single stage dynamic pressure assisted thermal spore inactivation.

Most of previous studies were conducted in buffers or food systems with low inlet temperature, and valve temperatures were not always monitored, which made it difficult to compare the reported results. While strong inactivation can be observed with higher homogenization pressures and inlet (and consequently valve) temperatures, the contribution of individual parameters was not clear and the influence of temperature on the inactivation should also be assessed. Furthermore, it should be noted that results of studies in model systems cannot be directly extrapolated to real food systems. The food constitutes could have an influence on the inactivation of microorganisms by UHPH as it was already shown for high isostatic pressure high temperature microbial inactivation (Georget et al., 2015). Some studies showed that milk fat could protect *Listeria monocytogenes* and endogenous flora against UHPH treatments (Kheadr et al., 2002; Vachon et al., 2002). Contrasting results were also reported in *Escherichia coli* suspended in buffers and milk (Diels et al., 2005; Briñez et al., 2006). Little information exists on the influence of milk fat on the inactivation of spores during UHPH treatments, and few comparative studies were found on the inactivation of spores by UHPH in model and food systems. While the investigations on vegetative microorganisms showed that no sublethal injury was observed after UHPH treatments (Wuytack et al., 2002; Briñez et al., 2007; Donsì et al., 2009; Roig-Sagués et al., 2009), there is a lack of information on the potential occurrence of such sublethal injury for spores following UHPH treatments.

When considering the efficacy of UHPH sterilization process, two important stress factors are underlined: pressure and temperature. It is thus expected that a good challenge strain should have a strong thermal resistance and potentially strong pressure resistance. Limited work was conducted on strains such as *G. stearothermophilus* and *C. sporogenes* spores (Pinho et al., 2011; Georget et al., 2014a) and showed that high thermal resistance is an essential attribute of potential surrogate for UHPH spore inactivation. In the present study, *Bacillus amyloliquefaciens* spores were used due to their resistance to high pressure thermal sterilization (Margosch et al., 2006; Sevenich et al., 2013) thus also potentially making them a challenge strain of interest. Spores suspended in PBS buffer (0.01 M, pH 7.0), low fat milk (pH 6.7), and whole milk (pH 6.7), were treated by a Stansted model FPG11300 UHPH unit. Following this, the UHPH inactivation profiles of spores suspended in PBS buffer and milk were compared. The influence of the fat content on the inactivation of spores was investigated. The thermal resistance of *B. amyloliquefaciens* spores was also assessed by modeling approaches to estimate the contribution of temperature to spore inactivation by UHPH.

## Materials and methods

### *B. amyloliquefaciens* spores preparation

The bacterial strain used in this study was *Bacillus amyloliquefaciens* FAD82 (Technische Mikrobilogie Weihenstephan, TMW 2.479), courtesy of Professor Michael Gänzle, University of Alberta, Canada. *B. amyloliquefaciens* spores were obtained following a method described elsewhere (Sevenich et al., 2013). A single colony of *B. amyloliquefaciens* grown on nutrient agar overnight was inoculated in 10 mL of nutrient broth. The suspension was incubated at 37°C and 250 rpm in a shaking incubator until the required optical density measured at a wavelength of 600 nm (OD_600_ = 1.6–1.8/control) was reached (controlled with a cell density meter Ultrospec 10, Amersham Biosciences GmbH, Germany). Subsequently, a volume of 200 μL of the culture was spread onto 2 × SG medium agar plates without antibiotics. After incubation at 37°C for 48 h, sporulation was monitored with a phase contrast microscope daily. When the percentage of sporulation reached more than 95%, the plates were stored at room temperature until the remaining vegetative cells were dried out. The spores were harvested using 4°C sterile distilled water and cleaned by repeated centrifugation (four-fold at 4800 g) until the supernatant was clean. The spore suspension was additionally treated twice with sonication for 1 min (35 kHz – 160 W_eff_) (Bandelin Sonorex RK 510H, Berlin, Germany) during the cleaning stage. The cleaned spore suspension at the concentration of ~10^8^ CFU/mL contained >95% phase bright spores without agglomerates as was verified by phase contrast microscopy and particle size distribution analysis (Mastersizer 2000, Malvern Instruments Ltd, Malvern, UK) (Table 1). Bacterial spores were stored in the dark at 4°C until use.

**Table 1:** **Particle size distribution analysis of *B. amyloliquefaciens* spores suspension (result transform type: volume)**.

**Parameters**	**Values**
Concentration	0.0018% Vol
Obscuration	6.38%
Weighted residual	5.221%
Specific surface area	6.35 m^2^/g
Uniformity	0.335
Span	0.978
Particle density	1.000
Mode	0.989
Surface weighted mean D [3,2]	0.946 μm
Volume weighted mean D [4,3]	1.130 μm
d (0.1)	0.670 μm
d (0.5)	1.023 μm
d (0.9)	1.670 μm

*B. amyloliquefaciens* spores were suspended in 0.01 M PBS buffer (137 mM NaCl, 2.7 mM KCl, 10 mM Na_2_HPO_4_ and 1.8 mM KH_2_PO_4_, pH 7.0) (Burston et al., 2008), low fat milk (1.5% fat, Gutesland, Netto Marken-Discount AG & Co. KG, Germany), and whole milk (3.5% fat, Gutesland, Netto Marken-Discount AG & Co. KG, Germany) at initial concentration of ~10^6^ CFU/mL. PBS buffer was selected because of its high stability at high temperatures and avoided potential pKa variation at high temperatures as was reported in previous work (Reineke et al., 2011b).

### UHPH treatment conditions

**Figure 1:**
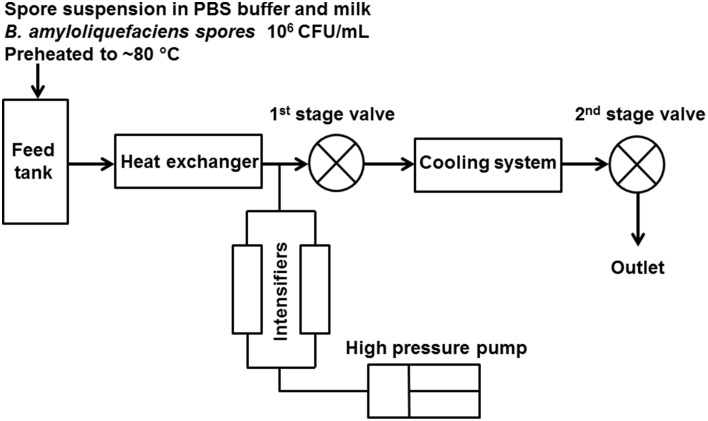
**Scheme of the UHPH unit setup (UHPH unit model FPG11300, Stansted Fluid Power Ltd, Harlow, UK)**.

The UHPH treatments were performed in a pilot-scale unit (model FPG11300, Stansted Fluid Power Ltd, Harlow, UK), and the scheme is shown in Figure 1. This device comprised two high pressure valves, the first of which is a ceramic valve able to support 400 MPa and the second of which maintains a slight back pressure of ~10 MPa. The spore suspension was preheated to ~80°C by recirculation through a tubular heat exchanger (DIL e.V., Quakenbrueck, Germany) and then processed through a plate heat exchanger (DIL e.V., Quakenbrueck, Germany) and the UHPH unit. The selection of the pre-heating temperature was based on previous work by the authors showing the absence of strong inactivation by UHPH for lower inlet temperatures at 37 and 55°C (Georget et al., 2014a). After the first valve, the temperature of the processing medium was decreased below 50°C within <1 s using glycol water at −10°C as the cooling agent. Samples were collected into sterile tubes and put in an ice bath immediately for subsequent plate counts. For UHPH conditions, the target inlet temperature was 80°C, and the pressures were 200, 300, and 350 MPa. The pressure fluctuation was no more than 10 MPa, except two trials for whole milk with treatments at 350 MPa, and the measured pressures were 366 and 370 MPa. The valve temperature was monitored, which was related to homogenization pressure and inlet temperature (~20°C of temperature increase per 100 MPa of pressure applied) and was used to compare to thermal inactivation kinetics based on an estimated residence time at valve temperature before cooling. The estimated residence times in different unit segments were derived from the measured flow rate under UHPH conditions (95.6 ± 4.2 L/h) and the equipment dimensions. All treatment conditions were repeated in three independent trials with the same initial spore batch.

### Thermal inactivation of *B. amyloliquefaciens* spores

To enable quasi isothermal conditions, thin wall glass capillaries were used to measure thermal inactivation kinetics of spores (Mathys et al., 2007b; Mathys, 2008) using the same spore batch as for the UHPH trials. Thin wall glass capillaries with an inner diameter of 1.0 mm, an outer diameter of 1.3 mm, and a length of 300 mm (Kleinfeld Labortechnik GmbH, Gehrden, Germany) were filled with a spore suspension volume of 100 μL. The capillaries were thermally treated in a thermostat (HaakeDC50, Karlsruhe, Germany) with silicon oil SIL180 (Thermo Fisher Scientific, Karlsruhe, Germany) as heating medium with temperatures ranging from 105 to 121°C and holding time between 15 and 7200 s depending on the temperature. After the defined holding time, the samples were rapidly cooled in an ice bath to avoid further inactivation. All thermal inactivations were repeated three times using the same spore batch.

### Microbiological analysis

Following thermal and UHPH treatments, samples were plated on nutrient agar (peptone 5.0 g, meat extract 3.0 g, agar 15.0 g, BD, Le Pont de Claix, France; distilled water 1000.0 mL, pH 7.0) in triplicate and incubated at 37°C for 48 h. The sublethal injury of spores was estimated by comparing colonies on nutrient agar alone and nutrient agar supplemented with 6% sodium chloride as a selective medium (NaCl). Preliminary experiments with different NaCl concentrations ranging from 3 to 12% showed that the maximum concentration that did not affect the growth of untreated *B. amyloliquefaciens* spores was 6%. Hence, both non-injured and injured spores would have the capacity to form colonies on nutrient agar medium while only non-injured spores could from colonies on the selective medium. Colonies were counted with a colony counter (Schuett-biotec, Göttingen, Germany). All inactivation graphs were obtained with Microcal Origin 8.0 (Microcal Software, Inc., Northampton, USA).

### Assessment of inactivation kinetic parameters

The inactivation of microorganisms has frequently been described by a first-order reaction. The reduction of microorganisms could be expressed as Equation (1) at a given temperature, where N_0_ is the initial spore concentration, and N_t_ is the corresponding viable number of spores at time t. The decimal reduction time D is the time required at a certain temperature to kill 90% of the microorganisms. It can be calculated from the semi logarithmic inactivation profile. The *z*-value is the temperature increase required to reduce the D value to a tenth of the original value. The rate constant k is obtained by Equation (2). The Arrhenius Equation (3) indicated the temperature dependence of the inactivation rate constant k.

However, thermal inactivation kinetics of *B. amyloliquefaciens* spores showed an initial lag phase (“shoulder”) which could not be sufficiently described by Equation (1), even when considering potentially different orders (Van Boekel, 2010). Another approach to model shoulder formations by assuming a non cultivable fraction of the initial population which would be activated by thermal shocks was also rejected (Abraham et al., 1990; Kessler, 2002). Based on the kinetics in this work, it would imply a very high speculated initial non cultivable population which was not observed by phase contrast microscopy. Thus, a Weibull model was also considered to assess the thermal inactivation kinetics. As shown in Equation (4), b is the scale factor and n is the shape factor (Peleg and Cole, 1998). The n parameter was not, or only slightly, dependent on temperature. It could be assumed as constant with varying temperatures, and an average value was calculated for modeling, which was 2.24, 2.22, and 1.84 for PBS, low fat milk, and whole milk, respectively. The temperature dependence of b could be expressed as the log-logistic model Equation (5), where T_c_ is the lethal temperature marker representing the temperature range where the inactivation accelerates and the lethality rate factor k is the rate representing the increase of b with temperature. This model was suggested by Corradini et al. (2005) and Periago et al. (2004) following work with *Clostridium botulinum* 213B and *Bacillus sporothermodurans* IC4 spores suspended in PBS and soups and was validated for thermal inactivation in the range of 101–125°C. According to this model, when T ≪ Tc, b ≈ 0 and when T ≫ Tc, b ≈ k(T-Tc) (Van Boekel, 2002).

The adjusted R^2^ given by Equation (6) is a modification of the correlation coefficient (R^2^). It indicates how well data points fit the curve, but it is adjusted for the number of parameters in a model and thus gives a more reasonable test of fit than R^2^ or the mean square error (MSE), especially when there are few data (n, number of data points) or a great number of parameters (N_T_) in the model (Davey, 1993; Khoo et al., 2003). When n ≫ N_T_,adjusted R^2^ approached the value of R^2^. The adjusted R^2^ allows for a comparative assessment of models with different numbers of parameters, which is important in this study.

(1) $$\log\left(\frac{{\text{N}}_{\text{t}}}{{\text{N}}_{0}}\right)=-\text{t}\cdot \frac{\text{k}}{\text{2}.\text{303}}$$

(2) $$\text{D}=\frac{\text{2}.\text{303}}{\text{k}}$$

(3) $$\text{ln}\left(\frac{\text{k}}{{\text{k}}_{0}}\right)=-\frac{{\text{E}}_{\text{a}}}{\text{R}}\cdot \frac{1}{\text{T}}$$

(4) $$\log\left(\frac{{\text{N}}_{\text{t}}}{{\text{N}}_{0}}\right)=-\text{b}\cdot t^{n}$$

(5) $$\text{b}=\text{ln}\left[\text{1}+\text{exp}\left(\text{k}\left(\text{T}-{\text{T}}_{\text{c}}\right)\right)\right]$$

(6) $$\text{Adjusted}{\text{R}}^{2}=1-\frac{\left(\text{1}-{\text{R}}^{2}\right)\left(\text{n}-\text{1}\right)}{\left(\text{n}-{\text{N}}_{\text{T}}-\text{1}\right)}$$

## Results

### Assessment of thermal inactivation kinetics

**Figure 2:**
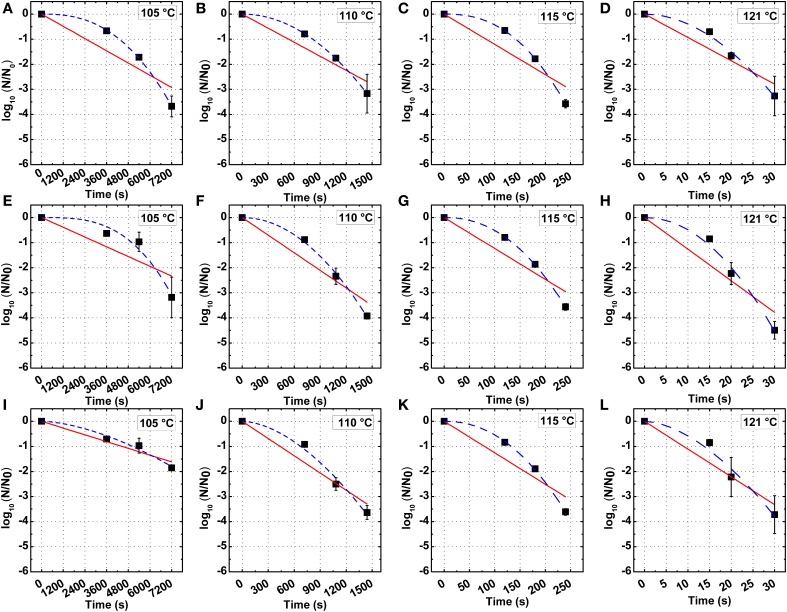
**First-order (red continuous line) and Weibull (blue dashed line) model of the inactivation kinetics of *B. amyloliquefaciens* spores in PBS (A–D), low fat milk (E–H), and whole milk (I–L) at 105, 110, 115, and 121°C**.

The thermal inactivation kinetics *B. amyloliquefaciens* spores in PBS and milk at 105, 110, 115, and 121°C are shown in Figure 2, with detailed kinetic parameters in Table 2. The decimal reduction time D, *z*-value and energy of activation E_a_ were evaluated based on the first-order reaction model.

**Table 2:** **First-order and Weibull thermal inactivation models' kinetic parameters (± followed by corresponding standard deviation)**.

**Medium**	**Temperature (°C)**	**First-order model**				**Weibull model**		
		**D (s)**	***z*-value (°C)**	**Ea (kJ/mol)**	**Adjusted R^2^**	**b**	**n**	**Adjusted R^2^**
PBS	105	2471.18 ± 211.18	7.02 ± 0.02	406.18 ± 1.27	0.88783	(1.31 ± 1.58) × 10^−9^	2.52 ± 0.22	0.99923
	110	542.69 ± 77.28			0.94176	(1.09 ± 1.08) × 10^−5^	2.01 ± 0.61	0.99987
	115	94.29 ± 19.40			0.89894	(1.29 ± 1.67) × 10^−5^	2.45 ± 0.42	0.99998
	121	11.90 ± 2.12			0.92851	(4.47 ± 2.54) × 10^−3^	1.96 ± 0.25	0.98724
Low fat milk	105	3039.33 ± 285.39	6.28 ± 0.13	454.22 ± 9.45	0.81162	(3.11 ± 4.40) × 10^−8^	2.62 ± 0.92	0.95477
	110	427.63 ± 24.07			0.93829	(2.01 ± 1.10) × 10^−6^	2.01 ± 0.09	0.99560
	115	81.28 ± 2.83			0.92379	(2.27 ± 1.36) × 10^−5^	2.20 ± 0.11	0.99978
	121	7.97 ± 0.63			0.91390	(5.51 ± 4.88) × 10^−3^	2.05 ± 0.26	0.98350
Whole milk	105	4474.11 ± 418.34	6.03 ± 0.10	473.35 ± 7.97	0.96538	(0.89 ± 1.51) × 10^−5^	1.72 ± 0.45	0.96463
	110	438.46 ± 33.45			0.95741	(1.26 ± 1.06) × 10^−6^	1.73 ± 0.01	0.97988
	115	79.73 ± 0.81			0.92804	(3.45 ± 2.40) × 10^−5^	2.17 ± 0.21	0.99946
	121	9.31 ± 2.05			0.94457	(1.86 ± 2.21) × 10^−2^	1.74 ± 0.44	0.96127

**Figure 3:**
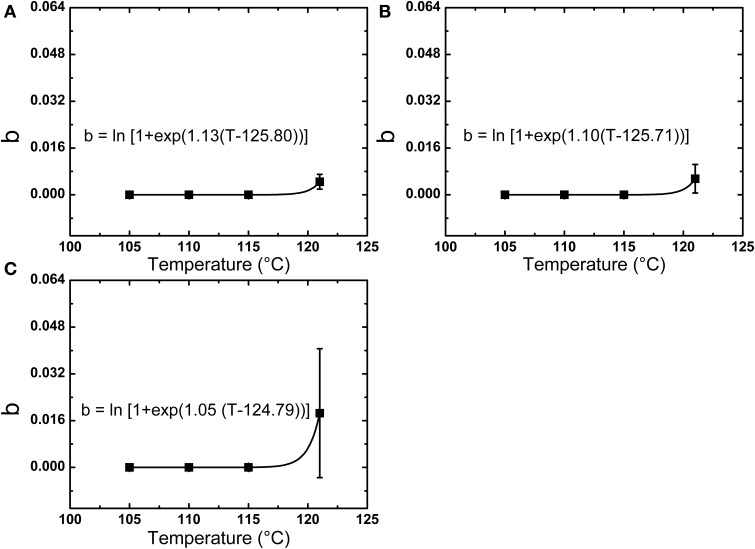
**Temperature dependence of the Weibull parameter b for the thermal inactivation of *B. amyloliquefaciens* spores in PBS (A), low fat milk (B), and whole milk (C)**.

A higher D value was found for spores in milk vs. PBS at 105°C, while at 110, 115, and 121°C, the D values in PBS or milk were not significantly different. The *z*-values of spores in PBS, lower fat milk, and whole milk were 7.02, 6.28, and 6.03°C, respectively. The E_a_ increased from 406.18 kJ/mol in PBS to 473.35 kJ/mol in whole milk, which suggested that the milk fat had some protective effect against thermal inactivation. One should however bear in mind that the overall fitting of the first-order reaction model to the experimental data was not optimal. In this sense, Weibull modeling offered a much better fitting. The adjusted R^2^ was determined to evaluate the accuracy of fitting models and showed that the use of a nonlinear model to describe *B. amyloliquefaciens* spore thermal inactivation is more appropriate from a statistical point of view. The b values increased with the increase of temperature, while the n values were slightly dependent on the temperature. Based on Equations (4) and (5), the b and n parameters were evaluated (Figure 3) and the modeled inactivation under higher temperatures, as such reached during UHPH, was calculated. There could be a risk of deviation from the real data in the extrapolation and it must be taken cautiously. However, it is important to point out that determination of the inactivation at *T* > 121°C was impossible experimentally because the very short holding times (<1 s) required to quantify the inactivation vs. time would not allow to reach the target temperature within the glass capillaries before full inactivation occurs (Mathys et al., 2007b).

### UHPH inactivation vs. pressure and valve temperature

**Figure 4:**
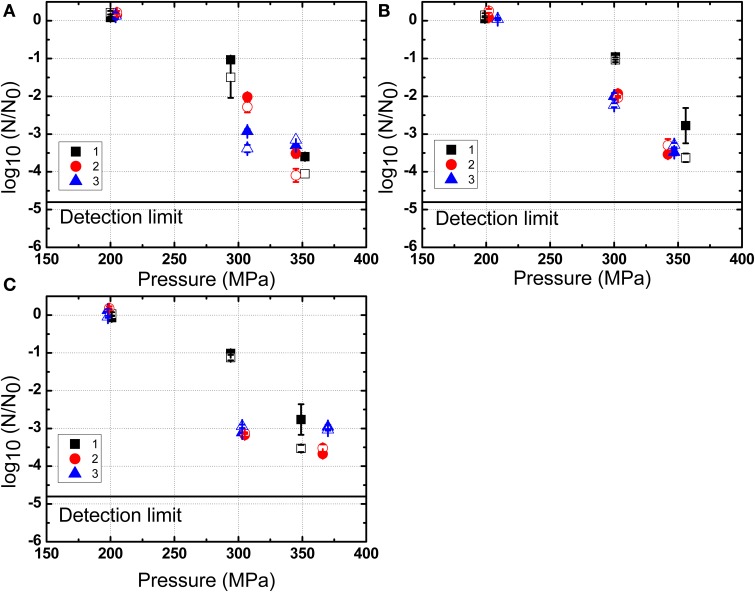
**Inactivation of *B. amyloliquefaciens* spores in PBS (A), low fat milk (B), and whole milk (C) as a function of the pressure**. 1, 2, and 3 represent three independent repetitions of the trial with the same initial batch of spores. The solid symbols represent results obtained by plating on nutrient agar medium and the corresponding hollow symbols are based on the use of the selective medium. The data shown are independent from the valve temperature.

**Figure 5:**
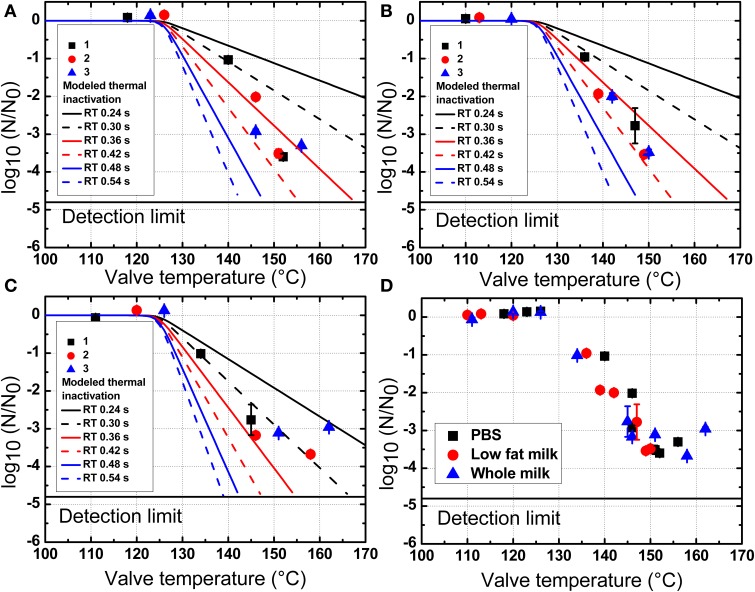
**Inactivation of *B. amyloliquefaciens* spores in PBS (A), low fat milk (B), and whole milk (C) as a function of the valve temperature**. All experimental plots are summarized in **(D)**. 1, 2, and 3 represent three independent repetitions of the trial with the same initial batch of spores. The modeled thermal inactivations are based on n values at 2.24 **(A)**, 2.22 **(B)**, and 1.84 **(C)** and b values calculated by equations in Figure 3. The data shown are independent from the homogenization pressure.

The inactivation of *B. amyloliquefaciens* spores in PBS and milk were plotted vs. the pressure (Figure 4) and the valve temperature (Figure 5). The valve temperature increased linearly with the increase of homogenization pressure at constant inlet temperature. This increase was the consequence of the intense energy conversion occurring at the first valve involving kinetic energy, turbulence, shear and cavitation forces (Hayes and Kelly, 2003; Thiebaud et al., 2003; Zamora and Guamis, 2015). It was reported that an increase of 22°C/100 MPa could be achieved at the valve of Stansted Fluid Power UHPH systems (Floury et al., 2004). In this study, temperature increase during UHPH treatments was similar and no significant difference was found between PBS buffer and milk.

As shown in Figure 4, the inactivation of *B. amyloliquefaciens* spores was enhanced with the increased pressure. No reduction of *B. amyloliquefaciens* spores was found at 200 MPa. Up to 3.5 log_10_CFU/mL reduction of spores was achieved at 350 MPa. The residence time after valve at high temperature and before cooling was estimated at 0.24 s based on the measured flow rate and equipment dimensions. Based on the experimental thermal inactivation of spores and extrapolation, different potential residence times ranging from 0.24 to 0.54 s were chosen to estimate the thermal inactivation cause by valve temperature during UHPH treatments. This range was selected to simulate potential deviations from the estimated mean residence time which are frequently reported for continuous processes. As shown in Figure 5, the increment in the valve temperature increased the spore inactivation. A valve temperature lower than 125°C during treatment at 200 MPa with an inlet temperature of 80°C led to no reduction of spores. With the increase of pressure, the valve temperature also increased and a higher spore inactivation was achieved. A reduction of 3.5 log_10_CFU/mL of *B. amyloliquefaciens* spores was achieved by treatment at 350 MPa with a valve temperature higher than 150°C and a very short residence time.

## Discussion

### Impact of the homogenization pressure on *B. amyloliquefaciens* spore inactivation

Based on Figure 4, it could be tempting to conclude that there is a linear correlation between homogenization pressure and inactivation. However, while this holds for high inlet temperatures, previous work (Georget et al., 2014a) also found that for lower inlet temperatures no inactivation could be achieved even for pressures as high as 350 MPa. Hence, the homogenization pressure appears to be a necessary facilitator for UHPH spore inactivation, but needs to be combined to high inlet and valve temperatures to yield a sufficient inactivation effect.

### Impact of the valve temperature on *B. amyloliquefaciens* spore inactivation

Inactivation of bacterial spores has commonly been modeled by a first-order reaction, even though a nonlinear inactivation curve was often observed. In this work, both log-linear and Weibull models were used to assess the thermal inactivation kinetics of *B. amyloliquefaciens* spores. Strong deviations to linear inactivation kinetics were observed and Weibull modeling provided a much better fit of experimental data, as was verified by the adjust R^2^ and could not be replaced by conventionally suggested alternative models (see Section Assessment of Inactivation Kinetic Parameters). Based on Weibull modeling and the determination of the temperature dependency of b Equation (5), the predicted thermal inactivation at very high temperatures/short holding times could be extrapolated to estimate the thermal contribution during UHPH treatments.

Previous studies showed that the inactivation of spores by UHPH increased with the increase of the inlet temperature. For *G. stearothermophilus* ATCC7953 and *C. sporogenes* PA3679 spores in skim milk, UHPH treatment of 16 passes at 300 MPa with inlet temperature at 45°C caused a reduction of only 0.67 log_10_CFU/mL (Pinho et al., 2011). No reduction of *B. subtilis* and *G. stearothermophilus* ATCC7953 spores was observed after treatment at 300 and 350 MPa with the inlet temperature at 37 and 55°C respectively. However, with inlet temperatures of 80°C, up to 5 log_10_CFU/mL *B. subtilis* PS832 spores and 2 log_10_CFU/mL *G. stearothermophilus* ATCC7953 spores were inactivated at 350 MPa (Georget et al., 2014a). This latter result also shows that the stronger resistance of a thermophilic strain and indicator for wet heat sterilization (*G. stearothermophilus* ATCC7953) correlated to its lesser inactivation by UHPH in comparison to a mesophilic strain such as *B. subtilis* PS832. It was also reported that treatment at 300 MPa with the inlet temperature at 85°C could completely inactivate *B. cereus, B. licheniformis, B. sporothermodurans, B. coagulans, G. stearothermophilus*, and *B. subtilis* spores in whole milk at the inoculating level of 6 log_10_CFU/mL. The *B. cereus* spores were below the detection limit for treatment at 200 MPa with the inlet temperature at 75°C and treatments at 300 MPa with inlet temperature at 55, 65, and 75°C (Valencia-Flores et al., 2013).

In this work, the overlapping of the predicted thermal inactivation kinetics and the experimentally observed UHPH inactivation indicated that in spite of the very short holding time before cooling takes place, the temperatures reached are high enough to induce significant thermal inactivation. The overlap of the predicted thermal and UHPH inactivation profiles suggests that the achieved inactivation of *B. amyloliquefaciens* spores was slightly higher than predicted based on thermal only inactivation with the estimated residence time of 0.24 s. UHPH inactivation profiles were closer to thermal inactivation profiles modeled with residence times of 0.36, 0.48, and 0.30 s in PBS, low fat milk, and whole milk, respectively. This could mean that either mechanic stresses at the valve additionally improved the inactivation post valve or that deviations to the estimated mean residence time occurred as can be observed in continuous systems with complex flow patterns (Georget et al., 2013). This latter hypothesis seems plausible, in particular when considering the very small range of residence times considered here (0.24–0.54 s). The modeled thermal inactivation profiles further emphasize the potential impact of very high temperatures on inactivation kinetics and the strong dependence on residence times, even for very short ones.

Previous work suggested that when valve temperatures below 60°C were reached during UHPH, microbial inactivation could be a synergetic action of all stress factors including cavitation, shear stress, turbulence, impingement and high pressure. However, when valve temperatures reached 80°C or above, the observed microbial inactivation was mainly caused by thermal effects (Pathanibul et al., 2009; Dumay et al., 2013; Georget et al., 2014b). This would also support the second hypothesis that deviations to the mean residence time might explain the slightly stronger spore inactivation observed by UHPH. Local recirculation zones before the cooling section could contribute to this behavior. Therefore, a proper characterization of the residence time distribution and temperature profiles for the considered valve design by computational fluid dynamic could increase the understanding of the key process parameters and potentially confirm the dominant role of temperature in bacterial spore inactivation by UHPH.

### Sublethal injury of *B. amyloliquefaciens* spores

No significant difference was observed in the colonies between selective and non selective media (Figure 4), indicating that the UHPH treatment apparently caused no sublethal injured spores. Inactivation by UHPH was often regarded as an “all or nothing” mechanism, which means no sublethal injury to the cells (Donsì et al., 2009). If the cells are disrupted into small fragments by the mechanical stresses, they are dead, otherwise, they are alive. There is no other physiological state in between. Existing studies found that no sublethal injury was caused in vegetative microorganisms by UHPH. *Yersinia enterocolitica* and *Staphylococcus aureus* were treated at 200 and 300 MPa with the inlet temperature at 25°C, and no sublethal injury was observed using low pH (5.5–7), NaCl (0–6%) or sodium dodecyl sulfate (SDS, 0–100 mg/L) as selective components in the plate medium. These findings differed from the high isostatic pressure processing that could result in an accumulation of sublethal injury, eventually leading to inactivation (Wuytack et al., 2002). Similar results were also found in UHPH treated *L. monocytogenens* in milk as well as *S. aureus* and *S. carnosus* inoculated in milk and orange juice (Briñez et al., 2007; Roig-Sagués et al., 2009). The same conclusion might apply to bacterial spores which are furthermore protected against mechanical stresses by their multi-layered and highly condensed structure. Hence, the inactivation mechanism in the matrices considered might be a binary one—once a certain stress threshold is reached, the spores are inactivated, and otherwise they stay physiologically fit.

### Protective effect of milk fat during UHPH treatments

Characteristics of food matrices (water and fat contents, viscosity, pH, etc.) have been found to influence microbial inactivation during UHPH treatments (Dumay et al., 2013). Some authors reported the milk fat could have a protective influence on vegetative microorganisms during UHPH treatments, in analogy to the well-established protective effect of milk fat during thermal treatments (MacDonald and Sutherland, 1993). Others suggested that fat might induce locally a stronger heating and thus improve inactivation (Roig-Sagués et al., 2009). UHPH treatments at 200 MPa with an inlet temperature at 28°C for 5 1-min cycles resulted in a reduction of 2, 3, and 4 log_10_CFU/mL of endogenous flora in full fat (3.5%), low fat (2%), and skim milk, respectively (Kheadr et al., 2002). The degree of inactivation of *L. monocytogenes* in milk was significantly lower than in PBS (Vachon et al., 2002). Milk being an oil-in-water emulsion, this protective effect could be explained by the interactions between fat globules and microorganisms. The size of fat globules decreased with the increase of homogenization pressure during treatments, and the particle size distribution was shifted toward smaller values. The volume weighted mean value D[4,3] decreased from 3.81 mm (control raw milk) to 0.189 mm after homogenization at 300 MPa (Picart et al., 2006; Dumay et al., 2013). Fat globules could offer local low water activity refuges to the microorganisms and thus provide potential protective effect against thermal inactivation.

However, contrasting results were also reported with the higher inactivation of *E. coli* in whole milk than in PBS and skim milk (Vachon et al., 2002; Briñez et al., 2006). It was also found that fat content increased the inactivation of *L. monocytogenes* in milk (Roig-Sagués et al., 2009). By evaluating the inactivation of *E. coli* in buffer solutions with different viscosities and food systems (skim milk, soy milk, and strawberry-raspberry milk), Diels et al. (2005) found that the inactivation of *E. coli* by UHPH was influenced by fluid viscosity, which was against the protective effect of milk fat.

In the present work, all experimental plots of *B. amyloliquefaciens* spores in PBS, low fat milk, and whole milk as a function of valve temperature are shown in Figure 5D. It could be observed that the milk fat had no clear protective effect on spores against UHPH treatments.

In conclusion, UHPH could be a promising alternative to high temperature sterilization for the preservation of pumpable and particle free foods. The modeled thermal inactivation matching UHPH treatment parameters suggests that the observed reduction in spore counts could be temperature driven in agreement with previous work by the authors. A reduction of 3.5 log_10_CFU/mL of *B. amyloliquefaciens* spores was achieved at 350 MPa with the valve temperature higher than 150°C for very short holding times. No sublethal injury was observed after UHPH treatments using sodium chloride as selective component in the nutrient agar medium. Future work based on advanced fluid dynamic modeling including exact residence time and temperature distributions could also allow refining the approach used in this work and assess whether stress factors such as turbulences or shear stresses could additionally contribute to thermal inactivation during UHPH.

### Conflict of interest statement

The authors declare that the research was conducted in the absence of any commercial or financial relationships that could be construed as a potential conflict of interest.
